# BMP2/BMPR1A is linked to tumour progression in dedifferentiated liposarcomas

**DOI:** 10.7717/peerj.1957

**Published:** 2016-04-19

**Authors:** Hannah L. O’Neill, Amy P. Cassidy, Olivia B. Harris, John W. Cassidy

**Affiliations:** 1 Aberdeen Royal Infirmary, University of Aberdeen, Aberdeen, United Kingdom; 2 Queen Elizabeth University Hospital, NHS Greater Glasgow and Clyde, Glasgow, United Kingdom; 3 Wellcome Trust-MRC Stem Cell Institute, University of Cambridge, Cambridge, United Kingdom; 4 Department of Pathology, University of Cambridge, Cambridge, United Kingdom; 5 Queens’ College, University of Cambridge, Cambridge Cambridgeshire, United Kingdom; 6 Institute of Cancer Sciences, University of Glasgow, Glasgow, United Kingdom

**Keywords:** Bone morphogenic protein, Bone engineering, Dedifferentiated liposarcomas, Soft tissue sarcomas, Bone repair, Orthopaedic surgery, Tissue engineering

## Abstract

Bone Morphogenic Protein 2 (BMP2) is a multipurpose cytokine, important in the development of bone and cartilage, and with a role in tumour initiation and progression. BMP2 signal transduction is dependent on two distinct classes of serine/threonine kinase known as the type I and type II receptors. Although the type I receptors (BMPR1A and BMPR1B) are largely thought to have overlapping functions, we find tissue and cellular compartment specific patterns of expression, suggesting potential for distinct BMP2 signalling outcomes dependent on tissue type. Herein, we utilise large publicly available datasets from The Cancer Genome Atlas (TCGA) and Protein Atlas to define a novel role for BMP2 in the progression of dedifferentiated liposarcomas. Using disease free survival as our primary endpoint, we find that BMP2 confers poor prognosis only within the context of high BMPR1A expression. Through further annotation of the TCGA sarcoma dataset, we localise this effect to dedifferentiated liposarcomas but find overall BMP2/BMP receptor expression is equal across subsets. Finally, through gene set enrichment analysis we link the BMP2/BMPR1A axis to increased transcriptional activity of the matrisome and general extracellular matrix remodelling. Our study highlights the importance of continued research into the tumorigenic properties of BMP2 and the potential disadvantages of recombinant human BMP2 (rhBMP2) use in orthopaedic surgery. For the first time, we identify high BMP2 expression within the context of high BMPR1A expression as a biomarker of disease relapse in dedifferentiated liposarcomas.

## Background

BMP2 is a member of the Transforming Growth Factor beta (TGF-*β*) superfamily of proteins and was first described for its essential role in the development of bone and cartilage (Chen, Zhao & Mundy, 2004). Recent studies have highlighted BMP2’s role as a multipurpose cytokine, involved in embryonic development (Munoz-Sanjuan & Brivanlou, 2002), migration (Song et al., 2014), angiogenesis (Song et al., 2011) and hedgehog signalling (Laforest et al., 1998). BMP2 signalling is essential for initiation of the endogenous bone repair response. Despite intact signalling cascades capable of directing osteogenesis, mice lacking BMP2 develop spontaneous fractures, which do not resolve without intervention (Tsuji et al., 2006). By combining osteoconductive hydrogel biomaterials with osteoinductive growth factors such as recombinant human BMP2 (rhBMP2), bone repair can be dramatically improved (Ben-David et al., 2013). Hence, there may be immediate clinical relevance in further annotation of the BMP2 signalling pathway in both normal tissues and in malignancy.

In order to initiate downstream SMAD signalling and the formation of the transcriptional complex, BMP2 binds type I and type II serine/threonine kinases at the cell surface. The current model suggests that type II receptors (BMPR2) can bind ligands in the absence of type I receptors but that type I receptors (BMPR1A and BMPR1B) are required for downstream signalling; likewise type I receptors require type II receptors for ligand binding (Bonor et al., 2012). Type I BMP receptors (BMPR1A and 1B) have well defined overlapping functions (Yoon et al., 2005) but may not be functionally redundant in all aspects of their biology. In the presence of ligand, a heterodimer forms between type I and type II receptors resulting in phosphorylation of BMPR1A/1B by BMPR2 and subsequent signal transduction through SMAD1/5/8 and SMAD4 (Bonor et al., 2012).

Despite BMP2s essential role in development there remains much debate over whether this translates to tumour progression (Gitelis, Wilkins & Yasko, 2008). Interestingly, a recombinant human version of BMP2 (rhBMP2) has found utility as a adjuvant in spinal surgery, but there remains concerns over its safety after heavy use was linked to new tumour formation (Carragee et al., 2013). Likewise, BMP2 has been linked to increased motility and invasiveness in gastric cancers (Kang et al., 2010). In the soft tissue sarcomas, BMP2 has long been suspected of playing a role in disease progression (Yoshikawa et al., 1994), but no definitive studies have been carried out. Herein, we sought to profile BMP type I receptor expression in soft tissue sarcomas in an attempt to explain the apparent dichotomy of BMP2 signalling. Specifically, we investigated the role of high BMPR1A expression within the context of high BMP2 expression in soft tissue sarcomas and found a significant association with reduced disease free survival. Through differential expression analysis and gene set enrichment, we show that this increase in disease recurrence is linked to increased extracellular matrix (ECM) remodelling in dedifferentiated liposarcomas. This study suggests that BMPR1A-biased BMP2 signalling leads to disease progression in soft tissue sarcomas and that this outcome is linked to ECM remodelling. Furthermore, our conclusions suggest that increased research into the effects of locally administered recombinant human BMP2 (rhBMP2) is needed, along with continued follow-up and screening of patients administered rhBMP2 during spinal surgeries.

## Methods

### Data acquisition

Protein expression data was downloaded from the Human Protein Atlas (Uhlén et al., 2010; Uhlén et al., 2015) (Cancer Atlas and the Cell Line Atlas; proteinatlas.org). Pan-cancer analysis was carried out using cBioPortal (cbioportal.org) (Cerami et al., 2012; Gao et al., 2013). With pathway data obtained from Cytoscape 3.2.1 (cytoscape.org) (Lopes et al., 2010). RNASeqV2 normalised gene results and clinical metadata were downloaded form the TCGA Data Portal (tcga-data.nci.nih.gov) (Sarcoma (provisional), Prostate (provisional), Breast (The Cancer Genome Atlas Network, 2012), Ovarian (The Cancer Genome Atlas Network, 2011), Lung (The Cancer Genome Atlas Network, 2014) and Head and Neck (The Cancer Genome Atlas Network, 2015)).

### Data analysis

Normalised RNASeqV2 expression values and corresponding clinical metadata were manipulated in R Studio (Mac) 0.99.484 (R Studio: https://www.rstudio.com/). Combined data were analysed in Microsoft Excel (Mac 14.4.3) and R Studio with results plotted in GrpahPad Prism 6 (Mac). Comparisons between two groups were made using two-tailed *t*-tests or Mann–Whitney *U*-tests and more than two groups by one-way ANOVA or Kruskal–Wallis, multiple comparisons were corrected for by Bonferroni *post hoc* tests. Kaplan–Meier disease free survival graphs were constructed in GraphPad Prism 6 and presented as whole population versus experimental population. Comparisons between curves were made by Log-rank (Mantel-Cox) tests with the Mantel-Cox *p* value reported.

### Differential expression analysis

For patient groups with observed differences in phenotype (pathway activation/disease free survival) differential expression analysis was performed on normalised RNASeqV2 gene counts. Data were combined in R studio and means across rows (genes) calculated. Multiple two tailed *t*-tests with Bonferroni correction were used to compare means. Using a false discovery rate of 0.05, differentially expressed genes were ranked based on log_10_ fold-change. Ranked genes were analysed through the Gene Set Enrichment pre-ranked plugin (GSEAPreranked at genepattern.broadinstitute.org) with default parameters and 1,000 permutations (GenePattern version 3.9.4 (Hubble et al., 2009)). Gene sets c2.all.v5.0.symbols.gmt (Curated) and c6.all.v5.0symbols.gmt (Oncogenic signatures) were used for comparison. Gene sets in the top 10 from the dataset based on enrichments score were considered relevant.

## Results

### Differential type 1 BMP receptor expression in normal tissues and sarcomas

Type I BMP receptors (BMPR1A and 1B) have well defined overlapping functions (Yoon et al., 2005) but may not be functionally redundant in all aspects of their biology. To begin to test this hypothesis, we first profiled expression of the type I and type II BMP receptors across normal tissues (Fig. S1). Whilst BMPR1A and BMPR1B showed similar expression across some tissues (for example heart, lung, skin, colon etc.) we observed differential expression in numerous tissues. For example, BMPR1B is highly expressed in all regions of the brain (cerebellum, hippocampus, cerebr BMP2/BMPR1A is linked to tumour progression in dedifferentiated liposarcomasal cortex and lateral ventricle) whereas BMPR1A has only moderate expression in the cerebral cortex, suggesting that BMPR1B signalling predominates in the central nervous system.

**Figure 1:**
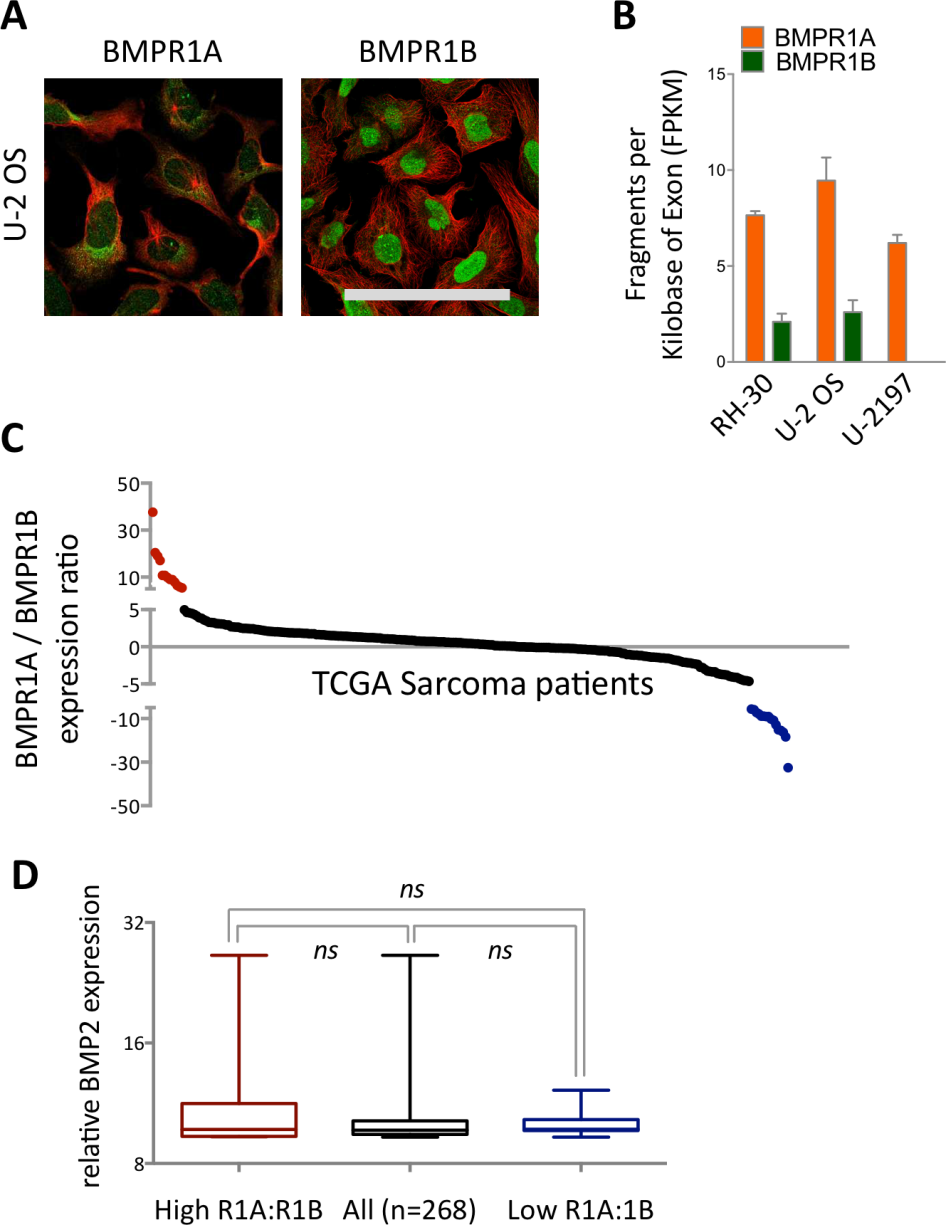
Differential expression and subcellular localisation of BMPR1A and BMPR1B. (A) Immunocytochemistry of BMPR1A and BMPR1B in U-2 OS osteosarcoma cell line. Green is receptor (Antibody CAB019398 for BMPR1A and HPA046821 for BMPR1B) red is microtubules. Scale bar is 100 µm. (B) RNAseq data from three sarcoma cell lines, RH-30 is a Rhabdomyosarcoma and U-2197 is a Malignant fibrous histiocytoma. (C) Ratio of BMPR1A to BMPR1B expression for each patient in the TCGA sarcoma dataset (*n* = 263). Patients with high BMPR1A:BMPR1B expression (top 10%) are colour coded red and patients with high BMPR1B:BMPR1A expression (top 10%) are colour coded blue. (D) BMP2 expression in patient sets defined in (A). Boxplot represents median with upper and lower quartiles with whiskers showing 95% confidence intervals, scale is log_2_.

We next profiled expression patterns across cancer cell lines (Fig. S2 and Fig. 1A). In the three cell lines profiled by immunohistochemistry (IHC), BMPR1A exhibited both nuclear and cytoplasmic localisation, which matched BMPR2 expression patterns (Fig. S2). In contrast BMPR1B appeared predominantly localised to the nucleus, but not nucleolus. The osteosarcoma cell line U-2 OS showed highest expression of BMPR1A by IHC (Fig. 1A), which was echoed in RNAseq data from three sarcoma cell lines (Fig. 1B), where BMPR1A expression far exceeded that of BMPR1B. These data suggest that BMPR1A and BMPR1B may not be functionally redundant in all aspects of their biology.

**Figure 2:**
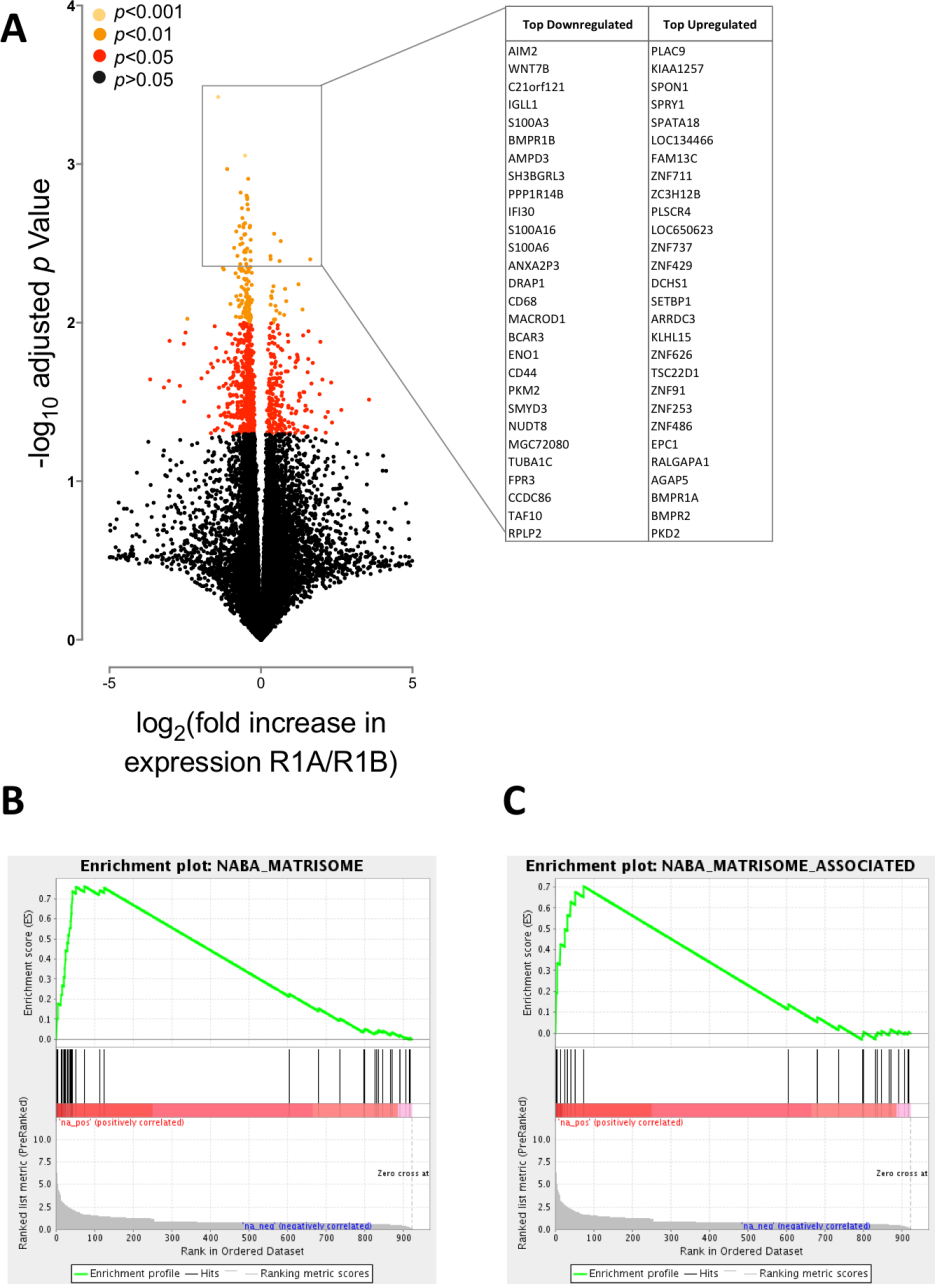
BMPR1A-biased BMP2 expression in soft tissue sarcomas. The results of differential expression analysis are shown comparing patients with high BMPR1A-biased and high BMPR1B-biased BMP2 expression i.e., upper quartile BMP2 expression and upper quartile R1A:B or R1B:A ratio. (A) volcano plot showing all differentially expressed transcripts colour-coded based on adjusted *p*-value (multiple two-tailed *t*-tests with Bonferroni correction post hoc). Insert shows top up- and down-regulated genes from this analysis. (B) GSEA enrichment plot for NABA Matrisome gene set; defined in silico based on lung and colon tumour extracellular matrix components (Naba et al., 2012). False discovery rate is *p* = 0.05. (C) GSEA enrichment plot for NABA Matrisome associated gene set. False discovery rate is *p* = 0.05.

As the largest differences in receptor expression patterns were observed in sarcoma cell lines and normal soft tissues, we elected to focus our analysis on BMP2 in the soft tissue sarcoma TCGA dataset. The observations from Figs. 1A and 1B (together with Figs. S1 and S2) that BMPR1A and BMPR1B have differential expression patterns led us to focus our analysis further on the effects of BMPR1A versus BMPR1B biased BMP2 signalling within this group of patients. As a proxy for this measure, we sought to identify patients with high BMP2 expression in the context of either high BMPR1A or BMPR1B expression.

By stratifying the 263 samples represented in the TCGA sarcoma dataset based on the ratio of BMPR1A to BMPR1B mRNA, we were able to define two clear groups (Fig. 1C). Although most patients expressed relatively similar levels of the two type I receptors there were clear groups who favoured either BMPR1A (red) or BMPR1B (blue) but who did not show differential expression of BMP2 (Fig. 1D). Based on the observed patterns of differential expression in normal tissues (Fig. S1) and cell lines (Fig. S2), and the conclusion that BMPR1A and BMPR1B may not be entirely functionally redundant, we reason that these two patient groups may show distinct outcomes to high BMP2 expression.

### BMP2/BMPR1A expression is associated with extracellular matrix remodelling

To test the hypothesis that patients favouring BMPR1A would have differential biological responses to high levels of endogenous BMP2 to patients favouring BMPR1B we refined our patient groups from Fig. 1C. Referred to as BMPR1A-biased or BMPR1B-biased BMP2, we defined groups of patients in the upper quartile of the BMPR1A/BMPR1B ratio and in the upper quartile of BMP2 expression together with their reciprocals (high 1B/1A with high BMP2).

We first asked whether BMPR1A-biased BMP2 expression led to differential gene expression to BMPR1B-biased. In total we found 922 differentially expressed genes out of a possible 20,533 annotated transcripts, using a false discovery rate (FDR) of 0.05 (corrected *p* < 0.05). Of these, 252 were up-regulated and 669 down-regulated. Fig. 2A shows a volcano plot relating fold change (in patients with BMPR1A-biased versus BMPR1B-biased BMP2 expression) to *p* value (multiple two-tailed *t*-tests with Bonferroni *post hoc*). The insert panel in Fig. 2A highlights a selection of the most differentially expressed genes; the full list is available as Table S1. Amongst the genes negatively associated with BMPR1A-biased expression are *WNT7B* which has a role in osteoblast differentiation and mineralised nodule formation (Shao et al., 2016), whilst positively associated is *SPON1* which inhibits excess bone formation (Palmer et al., 2014).

Although these data suggest that BMPR1A-biased expression is associated with reduced bone formation, which may be of importance to understanding rhBMP2 use in spinal surgeries, we sought to carry out a more unbiased analysis of our differential expression results in the form of a gene set enrichment analysis (GSEA). Figs. 2B and 2C show two of the most highly enriched gene sets, each associated with the matrisome, a computationally predicted collection of extracellular matrix (ECM) constituents and their regulatory networks (Naba et al., 2012).

Together, these data suggest that BMPR1A-biased BMP2 expression is associated with reduced bone formation and an increased turnover of ECM components. The interaction of tumour cells with ECM proteins can have profound effects on their biology, regulating gene expression, signal transduction and migration/invasion (Cassidy, 2014; Lu, Weaver & Werb, 2012). Moreover, BMP2 signalling has previously been linked to increased ECM production and degradation (Davidson et al., 2007). Thus, we next sought to determine whether BMPR1A-biased BMP2 expression had any clinical phenotype.

### BMPR1A-biased BMP2 expression is prognostic of poor clinical outcome

**Figure 3:**
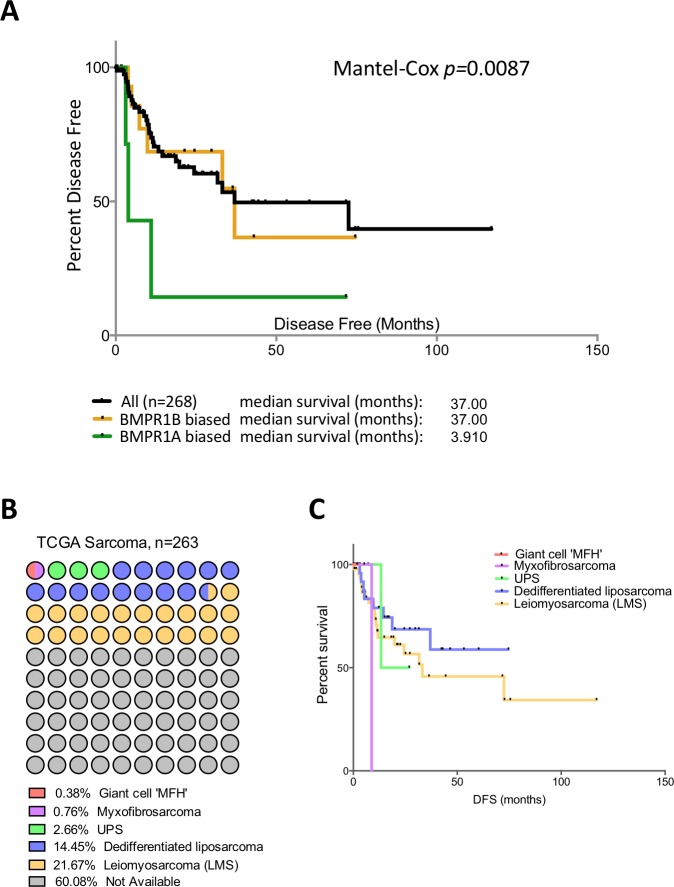
BMPR1A in the soft tissue sarcoma dataset. (A) Kaplan-Myer survival curves (disease free survival) for patients with BMPR1A or BMPR1B biased BMP2 expression. Median survival is shown. (B) Annotation of the TCGA sarcoma dataset with each dot colour-coded based on subtype and corresponding to 2.6 patients. The majority of the dataset was unclassified but can be expected to have similar proportions. (C) shows a Kaplan-Myer disease free survival curve for the different sarcoma subtypes, colours correspond to (B).

Patients in the TCGA sarcoma dataset have a median disease free survival of 37 months (Fig. 3A). Those patients with BMPR1B biased expression (*n* = 44) have no difference in their prognosis when compared to the dataset as a whole. However, those with BMPR1A-biased BMP2 expression (*n* = 32) have a significantly worse outcome when disease free survival is used as the primary endpoint (*p* = 0.0087). To discount prognostic influences of BMPR1A, BMPR1B or BMP2 expression individually, we constructed Kaplan-Myer curves for the entire sarcoma dataset and compared patients with the highest and lowest 10% expression of BMPR1A, BMPR1B, BMPR2, BMP2 and BMP7. Despite several interesting trends, no significant associations were seen (Fig. S3). Thus, in addition to ECM remodelling and reduced bone formation, soft tissue sarcoma patients with BMPR1A-biased BMP2 expression are more likely to show tumour progression in the presence of high levels of endogenous BMP2.

Soft tissue sarcomas are classified based on their origin in the soft but not connective tissues of the body. They can be separated based on their cell of origin and are generally considered a heterogeneous mixture of diseases (Cormier & Pollock, 2004). Figure 3B shows the TCGA dataset broken down into subtypes of soft tissue sarcomas. The two best-represented subtypes are Leiomyosarcoma (LMS; smooth muscle sarcomas) and Dedifferentiated liposarcoma (DLS; adipocyte sarcomas), though the majority (60%) of the dataset is unclassified. Disease free survival is shown in Fig. 3C; LMS tends to have a better prognosis than DLS though data for the three other subtypes is limited. To determine whether BMPR1A biased expression was localised to a specific subtype, we profiled expression of BMPR1A, BMPR1B, BMPR2 and BMP2 across the three best-represented subtypes (UPS, LMS and DLS). No significant differences were noted, though UPS did trend toward lower overall expression of both ligand and receptors (Figs. S4). The two largest groups, LMS and DLS had indistinguishable expression values of BMP signalling nodules. Interestingly, BMPR1A-biased BMP2 expression conferred a worse prognosis on patients than ether LMS or DLS. Thus, we next sought to determine whether the prognostic benefits of BMPR1A-biased signalling were split evenly across these two major subtypes.

### BMPR1A prognostic signalling is localised to dedifferentiated liposarcomas

**Figure 4:**
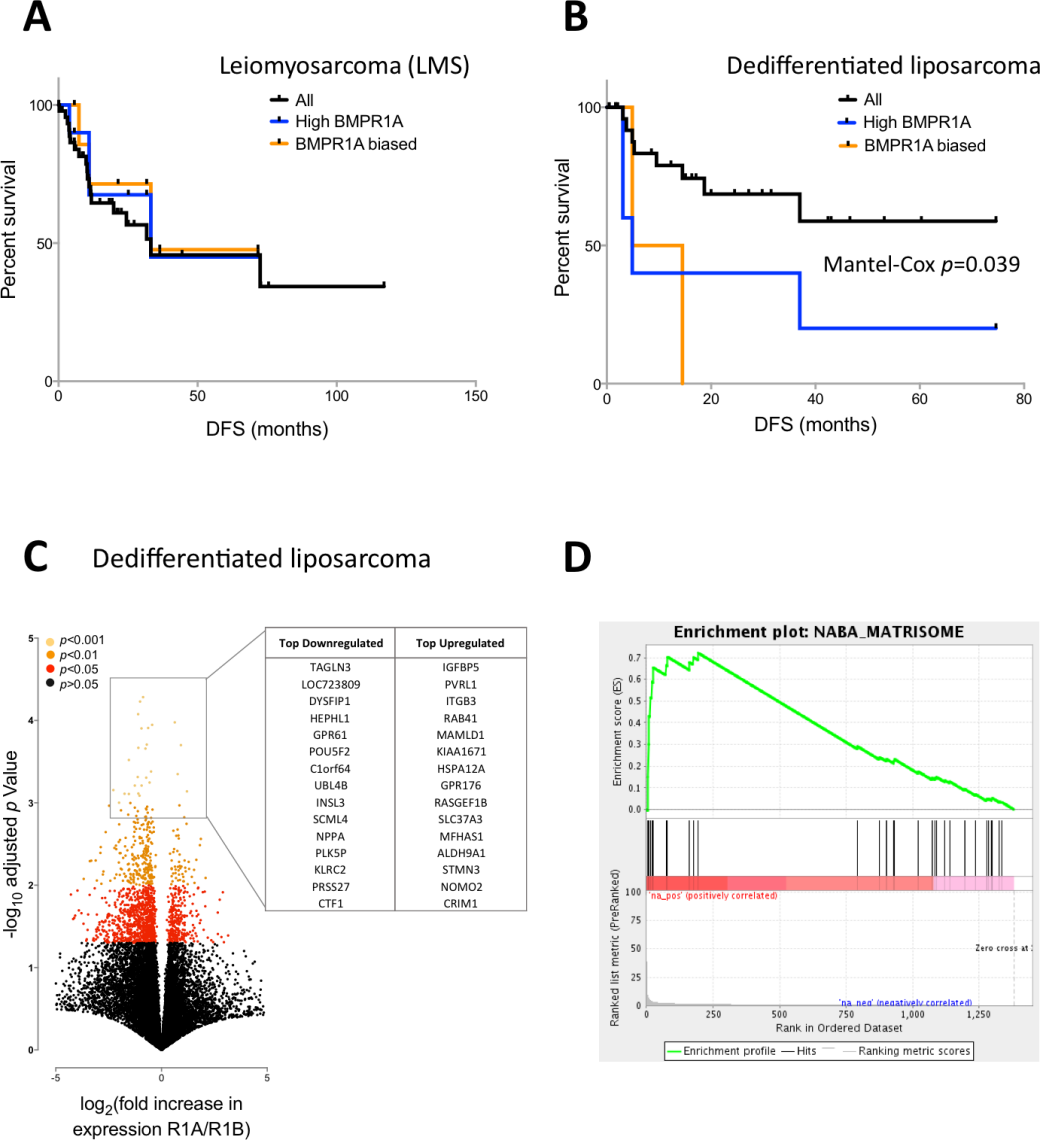
BMPR1A-biased BMP2 expression in DLS. Survival and differential expression analysis between patients with BMPR1A and BMPR1B biased signalling in dedifferentiated liposarcomas. (A) Disease free Kaplan-Myer survival analysis in Leiomyosarcomas for both high BMPR1A expression (top 10%) and BMPR1A-biased BMP2 expression (as defined previously). UPS is Undifferentiated Pleomorphic Sarcoma. (B) Disease free Kaplan-Myer survival in Dedifferentiated Liposarcomas for both high BMPR1A expression (top 10%) and BMPR1A-biased BMP2 expression. (C) Volcano plot comparing gene expression in Dedifferentiated Liposarcoma patients with BMPR1A-biased versus BMPR1B-biased BMP2 expression. Top up- and down-regulated genes are shown in the insert. (D) Gene set enrichment from differentially expressed genes based on a false discovery rate of 0.05. NABA Matrisome was the most significantly associated gene set. Defined in silico based on lung and colon tumour extracellular matrix components by Naba and collegues (2012).

We next profiled disease free survival in patients with LMS or DLS and investigated the effects of either BMPR1A-biased BMP2 expression or high BMPR1A expression alone. BMPR1A and BMPR1A-biased signalling appeared to have little effect in terms of disease free survival in patients with LMS (Fig. 4A), though high expression and R1A-biased BMP2 was highly detrimental in patients with DLS (Fig. 4B).

To investigate the mechanism for this effect, we compared global gene expression in DLS patients with BMPR1A-biased signalling versus those with BMPR1B-biased signalling. Despite working with a much reduced dataset of 10 versus 10 patients, we found 1,375 differentially expressed genes with a FDR of 0.05, the vast majority (1,061) of which were down-regulated (Fig. 4C); the full list is available as Table S2. Such a high number of differentially expressed transcripts compared to our early analysis in the whole sarcoma dataset (Fig. 2A) suggests patient groups which are more biologically robust. Amongst these differentially expressed genes, we noted a positive association between BMPR1A-biased BMP2 expression and GPR176, an orphan G-protein coupled receptor thought to be involved in atherosclerosis (Karagiannis et al., 2013). We performed GSEA on these differentially expressed genes and found a positive enrichment for a number of extracellular matrix and matrisome gene sets (Fig. 4D). These results further substantiate the observation that BMPR1A-biased BMP2 expression is associated with reduced bone formation and an increased turnover of ECM components as well as tumour progression.

Finally, we sought to determine whether the detrimental effects of BMP2-BMPR1A expression was a pan-cancer phenomenon. Several large studies performed by The Cancer Genome Atlas (TCGA) show copy number changes and mutational events in BMP2 interacting partners as defining features of that malignancy (Fig. S5). For example, two separate studies in Ovarian cancer show copy number gains as frequent events, which we later determined to be due to amplifications in the BMP2 and BMP7 loci (Fig. S6). Interestingly, mRNA up-regulation of both ligands and receptors were frequent events in each of the cancers profiled. Deep deletion of BMPR1A was a frequent event in prostate cancers, this has previously been shown to be related to the loss of the PTEN locus, which is associated with invasive forms of prostate cancer (Yoshimoto et al., 2012). In order to test whether our identified BMPR1A-biased BMP2 expression biomarker was useful across tumour types, we investigated disease free survival in Ovarian, Lung, Pancreatic and Prostate cancers, but found no significant alterations (Figs. S5). Thus, it appears that the detrimental effects of BMPR1A biased BMP2 expression are confined to the context of soft tissue sarcomas and in particular dedifferentiated liposarcomas.

## Discussion

Several studies have shown BMPR1A and BMPR1B have largely overlapping roles in BMP2 signal transduction, for example during chondrogenesis (Yoon et al., 2005). However recent work has highlighted opposing roles in certain situations. Sahni and colleagues (2010) found that BMPR1A, but not BMPR1B, knockout mice exhibited defective astrocytic hypertrophy and increased inflammatory infiltrate after spinal chord injury. The authors go on to show opposing roles for BMPR1A and BMPR1B in post-transcriptional regulation of astrocytic microRNA-21 (Sahni et al., 2010). We find that BMPR1A and BMPR1B expression is highly heterogeneous throughout the body without any obvious correlation between expression patterns. Moreover we observe differences in subcellular localisation in three separate cell lines. Together, these observations suggest that type I BMP receptors may not be functionally redundant, indeed differential expression of BMPR1A and BMPR1B may lead to differential outcomes of BMP ligand signalling in different tissue types. We reasoned that as BMPR1A expression is enriched relative to BMPR1B in sarcoma cell lines, BMP2-BMPR1A signalling may be important in progression of this tumour type.

Loss of BMPR1A has previously been associated with the formation of juvenile polyposis, a predisposing factor for gastrointestinal and colorectal cancers (Calva-Cerqueira et al., 2010). Reduced expression is also associated with a poor prognosis in pancreatic cancer (Voorneveld et al., 2013). Exemplifying the dichotomy of this signalling pathway in cancer, BMPR1A antagonists reduce growth and induce cell death in lung cancer cell lines (Langenfeld et al., 2013). Similarly, we find that sarcoma patients with BMPR1A biased BMP2 expression have significantly reduced disease free survival. Interestingly, we find that the prognostic value of BMPR1A biased BMP2 expression in sarcomas is localised to dedifferentiated liposarcomas and not leiomyosarcomas. Together these subtypes represent over 35% of the sarcoma dataset, with 60% undefined. As the trend holds true for the dataset as a whole, it is possible that other underrepresented subtypes (myxofibrosarcoma, giant cell sarcomas, undifferentiated pleomorphic sarcomas etc.) would show the same pattern if dataset annotation was complete, or if the dataset were larger.

Further to linking BMPR1A-biased BMP2 expression to reduced disease free survival, we report a detailed differential expression analysis in both the sarcoma dataset as a whole and dedifferentiated liposarcomas. Amongst the genes negatively associated with BMPR1A-biased signalling are *WNT7B* which has a role in osteoblast differentiation and mineralised nodule formation (Shao et al., 2016), whilst positively associated is *SPON1* which inhibits excess bone formation (Palmer et al., 2014). The ECM is far from an inert passenger to tumour progression. Rather, cells exist in a dynamic milieu of proteins, cytokines and growth factors which can have profound influence on the propensity to malignancy (Cassidy, 2014; Cassidy et al., 2014) and tumour heterogeneity (Cassidy, Caldas & Bruna, 2015). Indeed we find that patients with BMPR1A-biased BMP2 expression also exhibit increased expression of genes typically linked with the matrisome and ECM remodelling (Naba et al., 2012). Metalloproteinase expression has previously been linked to prognosis in soft tissue sarcomas (Benassi et al., 2001), and BMPR1A is necessary for extracellular matrix deposition by osteoblasts (Mishina et al., 2004). It is tempting to speculate that increased turnover of extracellular matrix in sarcomas is at least in part mediated by BMPR1A and this contributes to the observed poor prognosis of patients with BMPR1A-biased signalling. Together these data suggest a shift from an osteogenic phenotype to one of increased extracellular matrix remodelling.

In summary, we present here evidence that high expression of BMP2 leads to extracellular matrix remodelling and tumour progression in dedifferentiated liposarcomas, only within the context of high BMPR1B expression. Although this trend is present in the sarcoma dataset as a whole, we did not observe prognostic ability of BMPR1A signalling in four other solid tumours. rhBMP2 is used extensively as an adjuvant therapy for spinal fusion surgery and it is possible that such treatment could fuel progression of pre-existing sarcomas in these patients. Sarcomas are rare cancers and as such are underdiagnosed in the early stages, but this research underscores the need for continued study of the BMP2-BMPR1A signalling axis in cancer. Moreover, patients administered rhBMP2 should have substantial clinical follow-up to minimise the risk posed by any new cancer events.

## Supplemental Information

10.7717/peerj.1957/supp-1 Figure S1 Type1 receptor tissue expression Tissue specific protein expression of BMPR1A and BMPR1B based on IHC. Data from the Protein Atlas (Uhlen et al., 2010).

10.7717/peerj.1957/supp-2 Figure S2 Subcellular localisation of BMP receptors Immunostaining of BMP receptors in three cancer cell lines showing intracellular localisation. U-2 OS is an osteosarcoma, A-431 is an epidermoid carcinoma and U-251/MG is a glioblastoma astrocytoma line. Green is receptor (Antibody CAB019398 for BMPR1A, HPA046821 for BMPR1B and HPA049014 for BMPR2) red is microtubules. Scale bar is 100 µm.

10.7717/peerj.1957/supp-3 Figure S3 Prognostic affects of BMP pathway Kaplan-Myer disease free survival curves in the TCGA sarcoma dataset (*n* = 263) based on mRNA expression (top 10% and bottom 10% expressors). From top left; BMP2, BMP7, BMPR1A, BMPR1B and BMPR2.

10.7717/peerj.1957/supp-4 Figure S4 Receptor expression by sarcoma subset. Median (±interquartile range) expression of BMPR1A, BMPR1B, BMPR2 and BMP2 in different sarcoma subsets. UPS is Undifferentiated Pleomorphic Sarcoma and LMS is Leiomyosarcoma.

10.7717/peerj.1957/supp-5 Figure S5 BMP2 pan cancer (A) Pan-cancer analysis of BMP2 in several large scale genomics projects. Data from cBioPortal ( cbioportal.org) (Cerami et al., 2012; Gao et al., 2013). (B) BMP2 interacting partners, defined in Cytoscape 3.2.1 ( cytoscape.org) (Lopes et al., 2010).

10.7717/peerj.1957/supp-6 Figure S6 BMP2 pathway expression in several tumour types Detailed waterfall plots for BMP pathway members deregulated (mutation, copy-number, up/down-regulation) in various cancer types. Vertical bars represent individual patients from the following cancer types: Sarcoma (provisional), Prostate (provisional), Breast (Network, 2012), Ovarian (Network, 2011), Lung (Network, 2014) and Head and Neck (Network, 2015). Visualisation was cBioPortal (  cbioportal.org) (Cerami et al., 2012; Gao et al., 2013).

10.7717/peerj.1957/supp-7 Figure S7 Prognostic ability of BMPR1A signature in other solid tumours Kaplan–Myer disease free survival curves for BMPR1A-biased, BMPR1B-biased and BMPR2 signalling by high levels of endogenous BMP2. From top left, Prostate (provisional), Pancreatic (provisional), Lung (Network, 2014) and Ovarian (Network, 2011) cancers.

10.7717/peerj.1957/supp-8 Table S1 Differential expression analysis from BMPR1A-biased and BMPR1B-biased BMP2 signalling in the TCGA sarcoma dataset

10.7717/peerj.1957/supp-9 Table S2 Differential expression analysis from BMPR1A-biased and BMPR1B-biased BMP2 signalling in dedifferentiated liposarcomas

## Abbreviations

BMP2: Bone morphogenic protein 2
rhBMP2: recombinant human BMP2
BMPR1A: Bone morphogenic receptor type I A
BMPR1B: Bone morphogenic receptor type I B
BMPR2: Bone morphogenic receptor type 2
CRM: Compression-resistant matrix
DLS: Dedifferentiated liposarcomas
ECM: Extracellular matrix
GSEA: Gene set enrichment analysis
LMS: Leiomyosarcoma
TCGA: The cancer genome atlas
TGF-*β*: Transforming Growth Factor beta
UPS: Undifferentiated Pleomorphic Sarcoma

## Additional Information and Declarations

### Data Availability

The following information was supplied regarding data availability: The research in this article did not generate any raw data. All data were re-analysed from publicly available datasets (TCGA and Protein Atlas).
