# Supplementary figures and images for: BMP2/BMPR1A is linked to tumour progression in dedifferentiated liposarcomas
Source: PeerJ. 2016 Apr 19;4:e1957. doi: 10.7717/peerj.1957 (PMC4841227; doi:10.7717/peerj.1957)

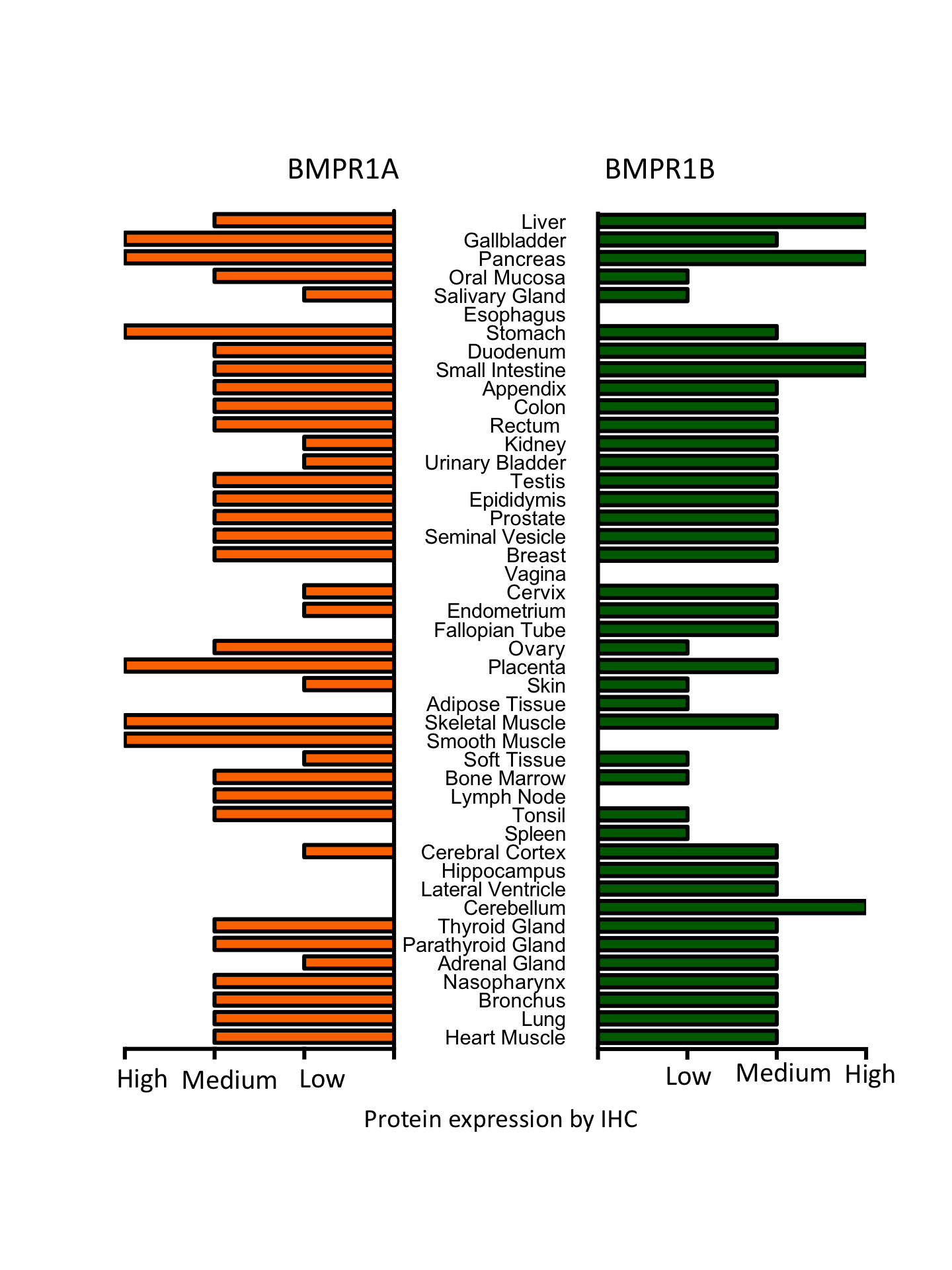

Supplement: Figure S1 — Tissue specific protein expression of BMPR1A and BMPR1B based on IHC. Data from the Protein Atlas (Uhlen et al., 2010). [file peerj-04-1957-s001.png]

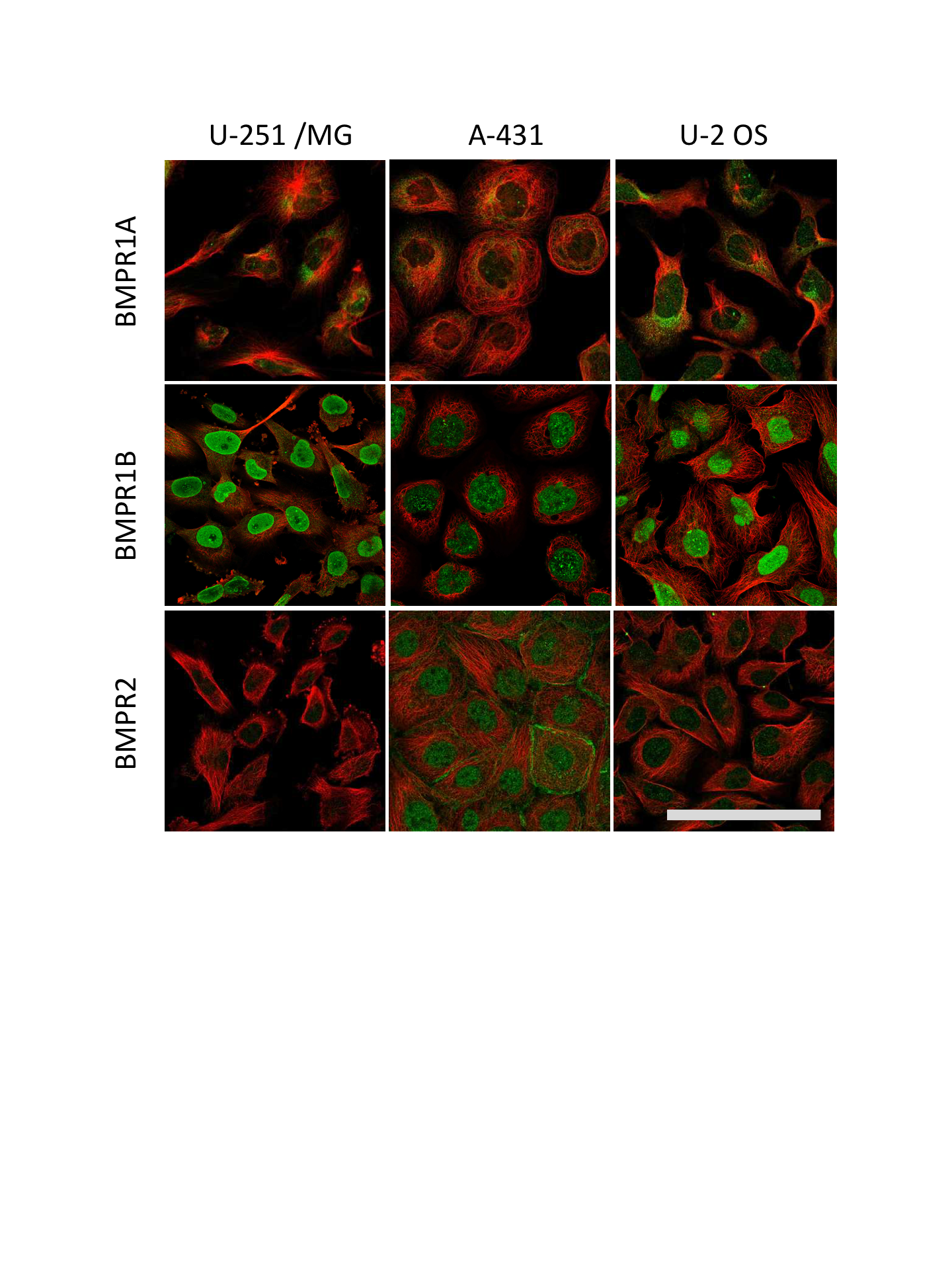

Supplement: Figure S2 — Immunostaining of BMP receptors in three cancer cell lines showing intracellular localisation. U-2 OS is an osteosarcoma, A-431 is an epidermoid carcinoma and U-251/MG is a glioblastoma astrocytoma line. Green is receptor (Antibody CAB019398 for BMPR1A, HPA046821 for BMPR1B and HPA049014 for BMPR2) red is microtubules. Scale bar is 100 µm. [file peerj-04-1957-s002.png]

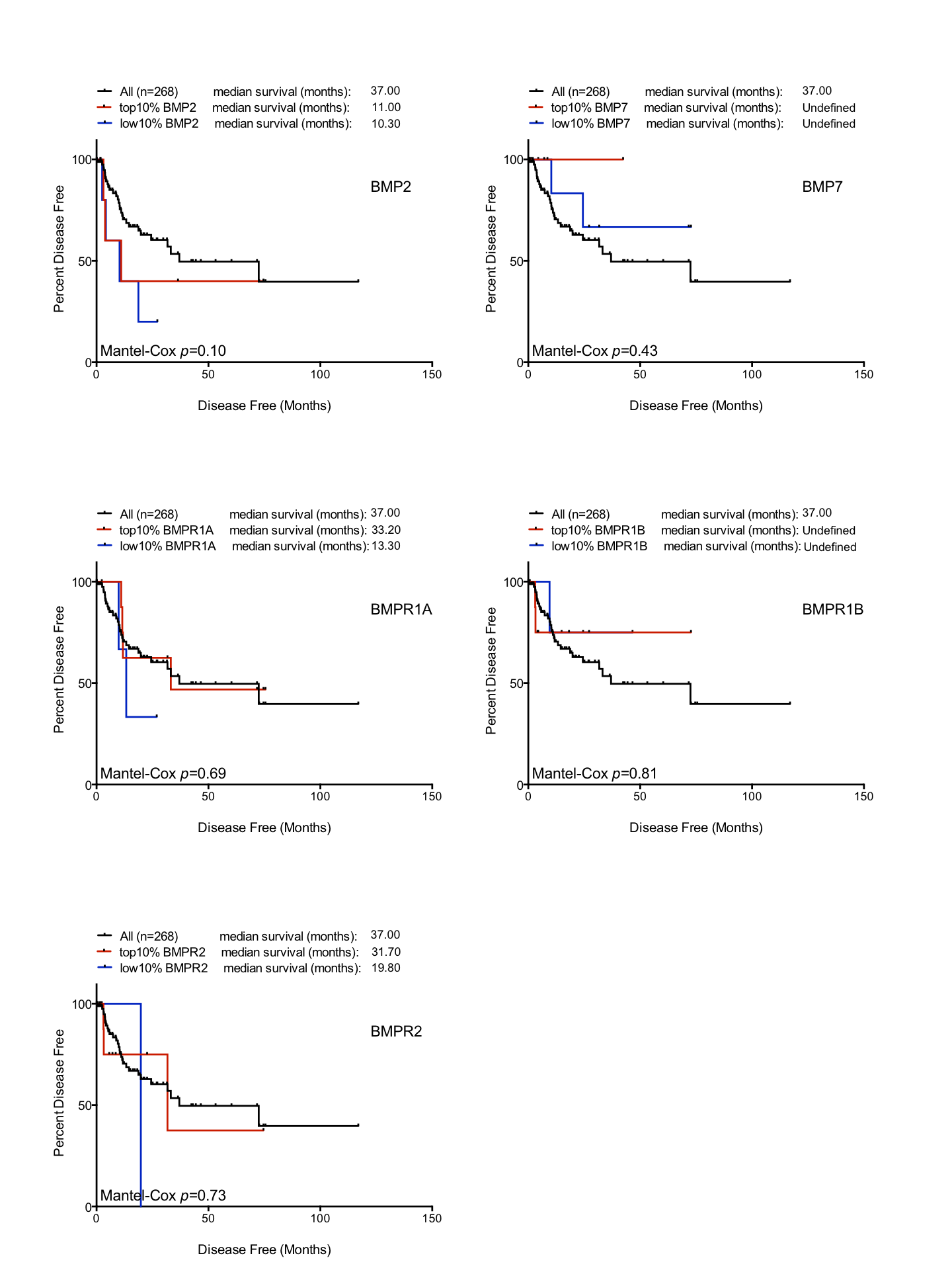

Supplement: Figure S3 — Kaplan-Myer disease free survival curves in the TCGA sarcoma dataset (n = 263) based on mRNA expression (top 10% and bottom 10% expressors). From top left; BMP2, BMP7, BMPR1A, BMPR1B and BMPR2. [file peerj-04-1957-s003.png]

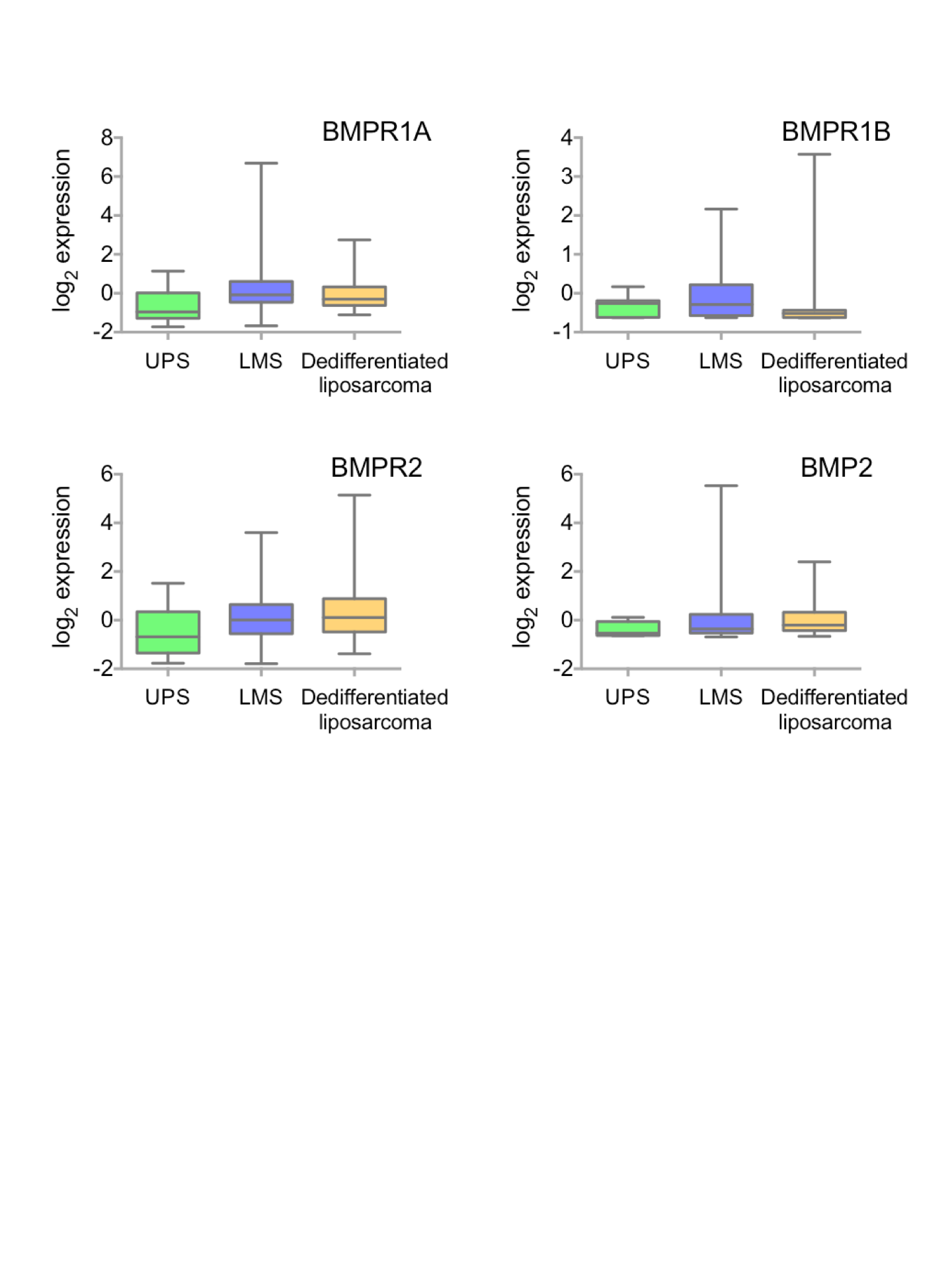

Supplement: Figure S4 — Median (±interquartile range) expression of BMPR1A, BMPR1B, BMPR2 and BMP2 in different sarcoma subsets. UPS is Undifferentiated Pleomorphic Sarcoma and LMS is Leiomyosarcoma. [file peerj-04-1957-s004.png]

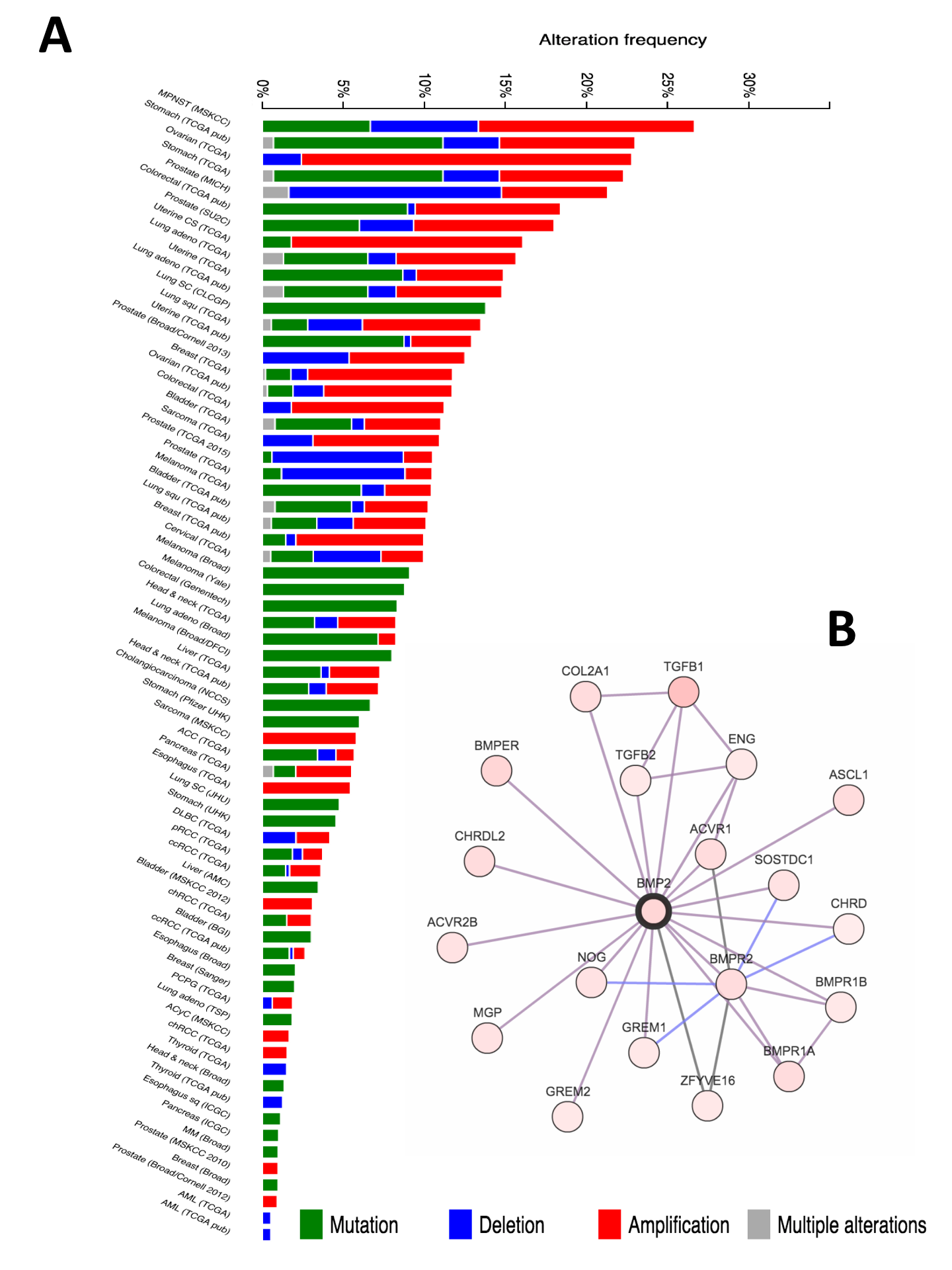

Supplement: Figure S5 — (A) Pan-cancer analysis of BMP2 in several large scale genomics projects. Data from cBioPortal ( cbioportal.org) (Cerami et al., 2012; Gao et al., 2013). (B) BMP2 interacting partners, defined in Cytoscape 3.2.1 ( cytoscape.org) (Lopes et al., 2010). [file peerj-04-1957-s005.png]

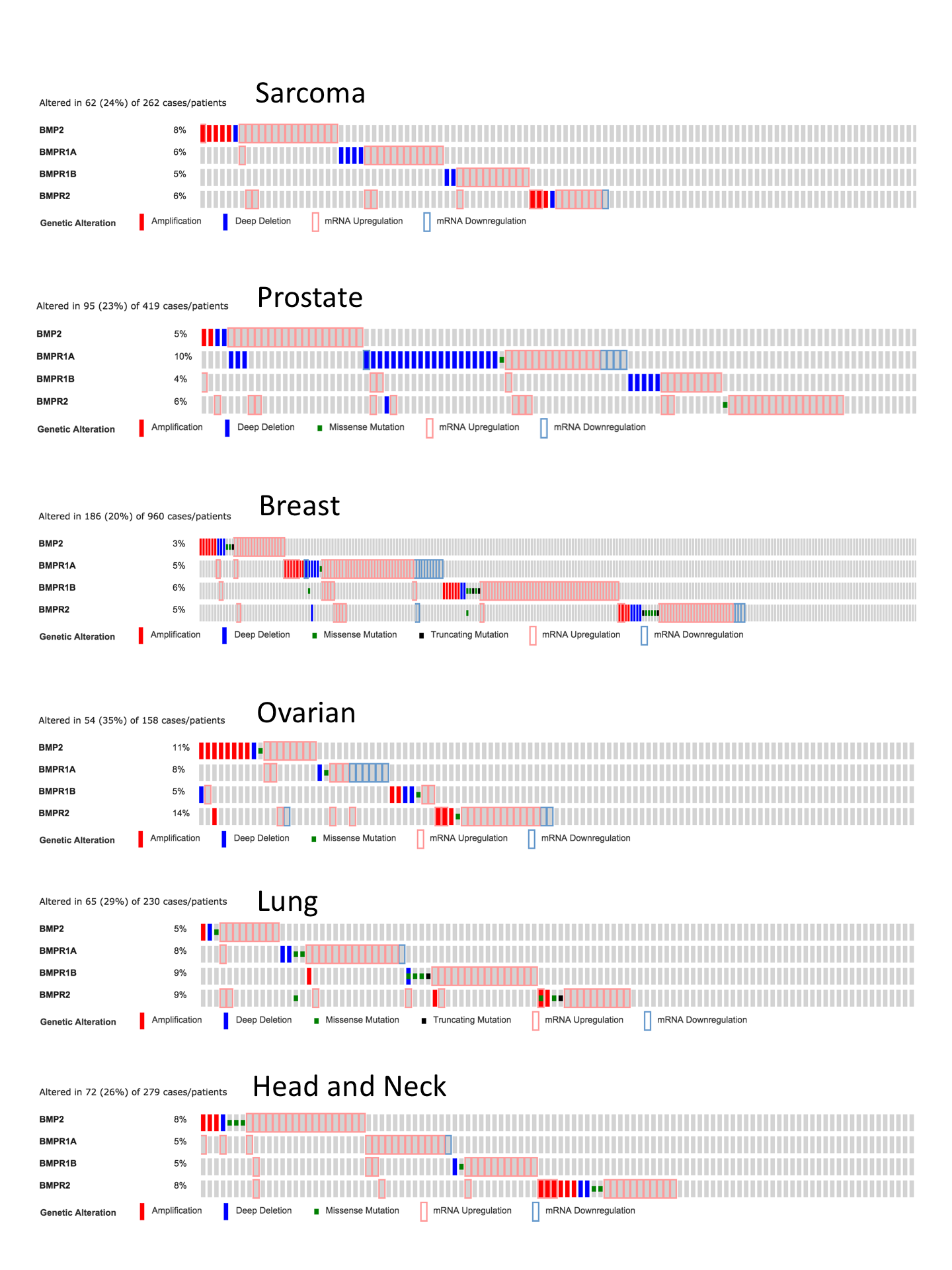

Supplement: Figure S6 — Detailed waterfall plots for BMP pathway members deregulated (mutation, copy-number, up/down-regulation) in various cancer types. Vertical bars represent individual patients from the following cancer types: Sarcoma (provisional), Prostate (provisional), Breast (Network, 2012), Ovarian (Network, 2011), Lung (Network, 2014) and Head and Neck (Network, 2015). Visualisation was cBioPortal ( cbioportal.org) (Cerami et al., 2012; Gao et al., 2013). [file peerj-04-1957-s006.png]

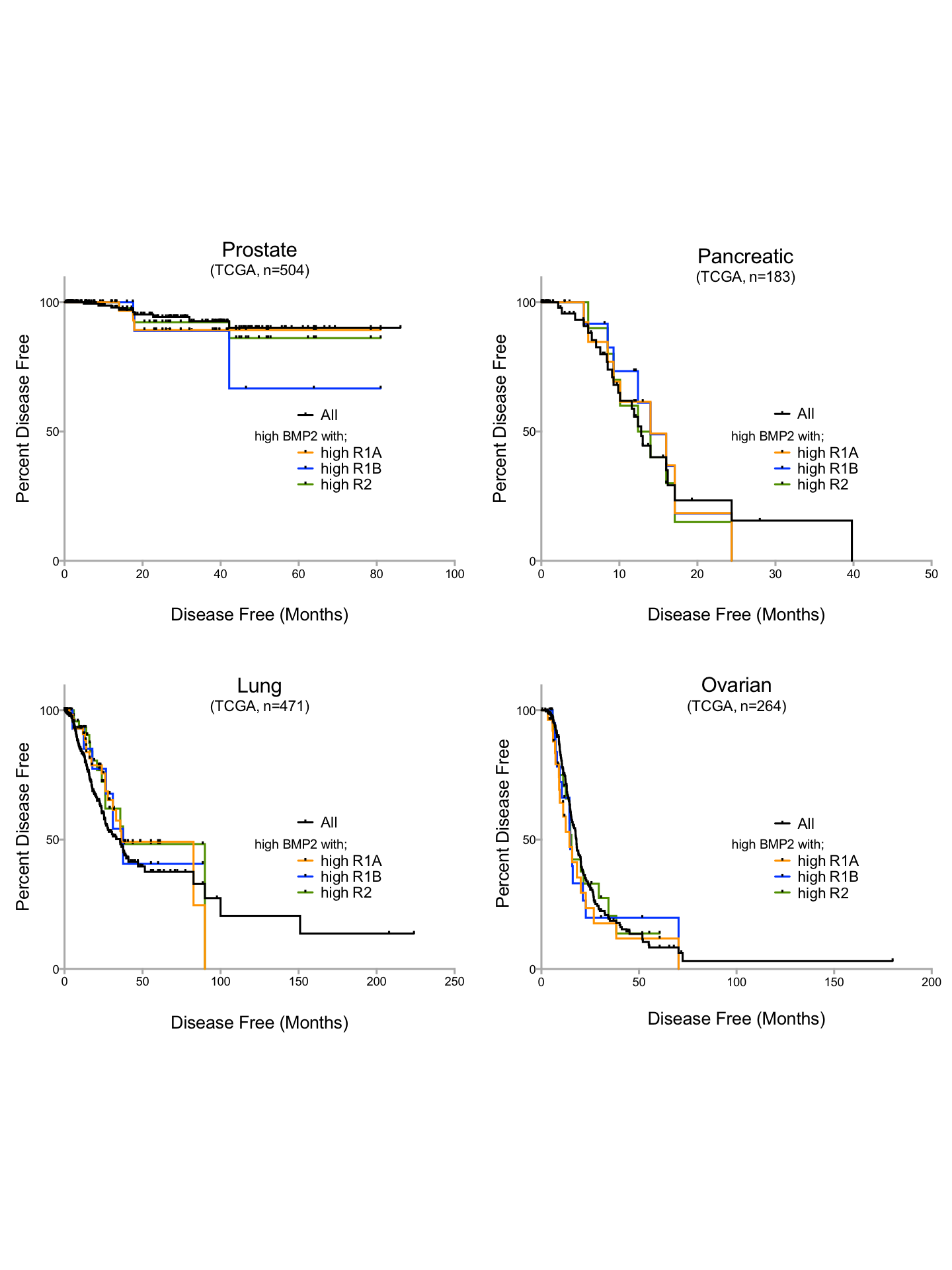

Supplement: Figure S7 — Kaplan–Myer disease free survival curves for BMPR1A-biased, BMPR1B-biased and BMPR2 signalling by high levels of endogenous BMP2. From top left, Prostate (provisional), Pancreatic (provisional), Lung (Network, 2014) and Ovarian (Network, 2011) cancers. [file peerj-04-1957-s007.png]
